# Effects of a health education intervention on hypertension-related knowledge, prevention and self-care practices in Nigerian retirees: a quasi-experimental study

**DOI:** 10.1186/s13690-019-0349-x

**Published:** 2019-05-23

**Authors:** Eyuche L. Ozoemena, Cylia N. Iweama, Olaoluwa S. Agbaje, Prince C. I. Umoke, Osmond C. Ene, Perpetua C. Ofili, Benedicta N. Agu, Charity U. Orisa, Michael Agu, Enejoh Anthony

**Affiliations:** 1 Department of Human Kinetics and Health Education, Faculty of Education, University of Nigeria, Nsukka, Enugu State, Nigeria; 2 Department of Public Health, Faculty of Health Sciences, Madonna University Elele, Port Harcourt, Rivers State Nigeria; 3 Department of Human Kinetics, Health and Safety Education, Ignatius Ajuru University of Education, Port Harcourt, Rivers State Nigeria

**Keywords:** Hypertension, Intervention, Health education, Knowledge, Prevention, Self-care

## Abstract

**Background:**

Education is vital to increasing knowledge, improving prevention and self-care practices for hypertension in older adults. This study aimed to determine the effectiveness of a health education intervention in improving hypertension (HT) knowledge, prevention and self-care practices among retirees in Enugu State, South-east, Nigeria.

**Methods:**

In this quasi-experimental study, we enrolled 400 participants in Enugu and Nsukka cities in Enugu State, south-east Nigeria. Participants were assigned to the treatment and control groups. Participants in the intervention/treatment group (T-group) received the intervention provided by public health experts and nurses and participants in the control group (C-group) received health talk without the intervention. Data collected at baseline (before intervention), 16 weeks (4th month) and follow-up (5th month) included demographic variables, knowledge about hypertension, prevention and self-care practices. We used paired samples *t*-test, Chi-square test and one-way ANOVA repeated measures for data analyses.

**Results:**

The mean age of the participants was 65.9 (± 8.9) years, the mean SBP and DBP were 136.5 (± 13.3) and 87.9 (± 9.1) respectively. More than half of the participants were (50.3%) were males, and the mean BMI was 23.9 (± 5.1) kg/m^2^. The paired comparison analysis showed that the mean HT knowledge score significantly increased in the T-group between baseline and 1 month (4th month) post-intervention compared to those in the C-group (*P* < 0.0001). Also, PA (*P* = 0.007), sleep pattern and quality (*P* = 0.003), substance use abstinence (*P* = 0.000), healthy diet (*P* = 0.000), and medication adherence (*P* = 0.000) improved significantly in the T-group compared to the control between baseline and 1 month after intervention. The repeated measures analyses showed statistically significant effects (between-groups analysis) for all outcomes with small to large effect sizes. Similarly, the repeated measures ANOVA analyses showed significant time-by-group interaction effects (within-groups) for all the outcomes with small to large effect sizes.

**Conclusion:**

Community-based health education intervention targeted at older adults can increase HT knowledge, improve prevention and self-care practices of hypertension at the population level.

**Electronic supplementary material:**

The online version of this article (10.1186/s13690-019-0349-x) contains supplementary material, which is available to authorized users.

## Background

Hypertension is a major modifiable risk factor for cardiovascular disease (CVD) and premature mortality globally [1–3]. Hypertension (HT) contributes to the burden of heart disease, stroke, kidney failure, premature mortality, and disability. Considerable evidence from observational studies has revealed strong, positive relationships between hypertension and risk of CVD and mortality [3, 4]. It is predominantly prevalent among diverse populations in developing nations where the health systems are weak [5]. It is estimated that hypertension affects about 1 billion people globally [6, 7]. The prevalence of hypertension is highest in the African Region at 46% of adults aged 25 years and above, while the lowest prevalence at 35% is found in the Americas [5].

Nigeria is the most populous country in Africa with an estimated population of 191 million (51% male, 49% female) with an estimated growth rate of 2.43% per annum and a high dependency ratio of 88% [8]. Due to this vast population, Nigeria immensely contributes to the total burden of hypertension in the continent [6, 9, 10].. The prevalence of hypertension in the adult population in Nigeria is increasing, although, the prevalence rate varies yearly and regionally [11–13]. For instance, a number of systematic reviews and meta-analysis conducted by Adeloye et al. [6] showed an estimated overall hypertension prevalence of 28.9% while Akinlua et al. [11] and Ogah et al. [13] reported that HBP prevalence ranged from 2.1 to 47.2%, and 8–46.4% in adults respectively.

Research has indicated that hypertension is more prevalent among the elderly population due to its higher prevalence and associated morbidity and mortality in this age group [3]. The poor health outcomes associated with uncontrolled hypertension include stroke, dementia, heart failure, myocardial infarction and renal failure [14]. Advancing age, an unmodifiable risk factor for high blood pressure (HBP) or hypertension has been linked with a progressive increase in the risk of vascular mortality with a 20 mmHg rise in SBP above 125 mmHg or 10 mmHg above DBP of 75 mmHg [3, 15]. Also, demographic characteristics and lifestyle factors such as gender, family history of CVD, unhealthy food intake, physical inactivity, tobacco and alcohol use, abnormal serum lipids and lipoproteins, obesity, chronic stress, and insufficient sleep are also strong risk factors for hypertension in adult populations [16–18].

However, evidence suggests [19, 20] that most individuals suffering from hypertension, especially the elderly are unaware of its presence, thereby increasing the occurrence of associated complications. Knowledge or awareness of hypertension is a strong predictor of prevention practices, treatment and medication adherence among hypertension patients [21–23]. A Nigerian study reported that only about 30% of hypertensive patients was aware of their condition at the time of diagnosis [24]. Also, prevention of hypertension and its comorbid conditions, modifications in medication adherence, lifestyle changes that depend on an in-depth comprehension of knowledge-based awareness have been suggested in literature [22]. Effective control of hypertension through valid health interventions has been shown to decrease the risk of cardiovascular complications especially that of systolic blood pressure (SBP) which is more prevalent among the elderly population [14, 19, 25]. In addition, effective self-care behavior or practice can facilitate cut back cardiovascular disease complications, however the speed of engagement in self-care practice is comparatively low among older patients including retirees [26].

Hence, it is imperative to evaluate the effectiveness of a community-based health education intervention on improving the HT knowledge, prevention and self-care practices of retirees in Enugu, south-east Nigeria. Studies have shown that older adults including retirees constitute a high-risk group for hypertension [3, 15–18]. The transition to retirement has been identified as important phase of life [27].. Van Dyck et al. [27] further opined that early retirement offers chances to imbibe a healthy lifestyle, and new habits can be developed by retirees due to available ample time previously spent working [28]. Also, literature suggests that retirees appear to be receptive to behavioural change [29].

Implementing an effective community-based educational intervention to improve knowledge and prevention of risk factors becomes necessary [30]. To our knowledge, no previous community-based HT education programme evaluation is reported for retirees in Enugu, south-east Nigeria. Hence, we hypothesized that a well-designed, validated, and culturally contextualized community-based health education intervention targeting retirees could lead to increased knowledge, improved HT prevention and self-care practices in the treatment group relative to the control group.

## Materials and methods

### Study design, setting and population

This study was a community-based quasi-experimental conducted from June to November 2018 in Nsukka and Enugu, Enugu State. According to the national census of 2006, the estimated population of Enugu state was 3,267,837. It comprised 1,596,042 males and 1,671,795 females [31]. The state has 18 Local Government Areas (LGAs) while Enugu is the central city. At the time of the study, 9613 retirees had fulfilled the mandatory 35 years in public service or attained the retirement age of 60 years as stipulated by Enugu State Civil Service Commission [32, 33]. The retirees are members of the Nigeria Union of Pensioners (NUP) and registered with the Enugu State Ministry of Finance.

### Sample size and determination procedure

This quasi-experimental study aimed at evaluating the effectiveness of health education intervention in increasing the HT knowledge, prevention and self-care practices among retirees as primary outcome variables. We calculated the study sample size based on a comparison of two independent means formula with an effect size of 0.33 and a standard deviation of 3.0. We set the level of significance at *p* < 0.05, and the study power was assumed to be 80% [34]. As a result, the estimated sample size was 286 participants (i.e., *n*_1_ = 143, *n*_2_ = 143). Since multivariable analyses were conducted for body mass index (BMI), BP levels, HT knowledge, prevention and self-care practices, other sociodemographic variables, and considering the possibility of respondents’ drop-outs, we decided to increase the number of study participants 40% more than the estimated sample size [35]. As a result, the calculated sample size was 286 + 114 = 400. Thus, we selected 400 subjects for the study. We used a two-stage sampling procedure for sample selection. We purposively selected three pension zones with the highest number of retirees in Enugu State at the time of the study [33] to enhance representativeness. These comprised Enugu (*n* = 6363), Abakpa (*n* = 1228), and Nsukka (*n* = 1137). Convenience sampling was used to select the study subjects. Subsequently, we randomly assigned 200 subjects each to the experimental group (*n*_1_ = 200) and control group (*n*_2_ = 200). The experimental or treatment and control groups consisted of 100 each of men and women. The treatment and control groups were located in Enugu and Nsukka respectively to avoid contamination. We estimated the statistical power using the G*Power 3.1.9.2 software version developed by Faul et al. [34]. We employed the *priori* power analysis for the difference between two independent means (two groups) procedure. We used the conventional value of *α* = 0.05 as indicated by Hickey et al. [35]. The results of statistical power analysis showed that the critical z = 1.96835, corresponding to α = β = 0.8020829 (Additional file 1). The results showed that the minimum sample size was enough for the study. The inclusion criteria included retirees aged ≥60 years who gave informed consent and located within the selected zones. We excluded retirees who were ill prior to the study and those who declined participation.

### Recruitment and data collection

We requested the leaders/chairpersons of the retirees in the selected pension zones to inform their colleagues about our study, rationale for the study and need for their involvement in the study. However, we emphasized voluntary participation by the retirees. Subsequently, we agreed on a specified date and time based on mutual consent. The leaders selected the weekly meeting days. When we arrived at the designated/specified venues, the leaders introduced the principal investigators and other research assistants to the retirees. Thereafter, we explained the details of our research, and asked if they agreed to participate in the study. All the retirees present at the venues agreed to participate in the study. Next, we gave the retirees a brief on the educational programme and administered the pre-test. The data collection process was conducted in Enugu and Nsukka venues.

### Educational intervention

**Fig. 1:**
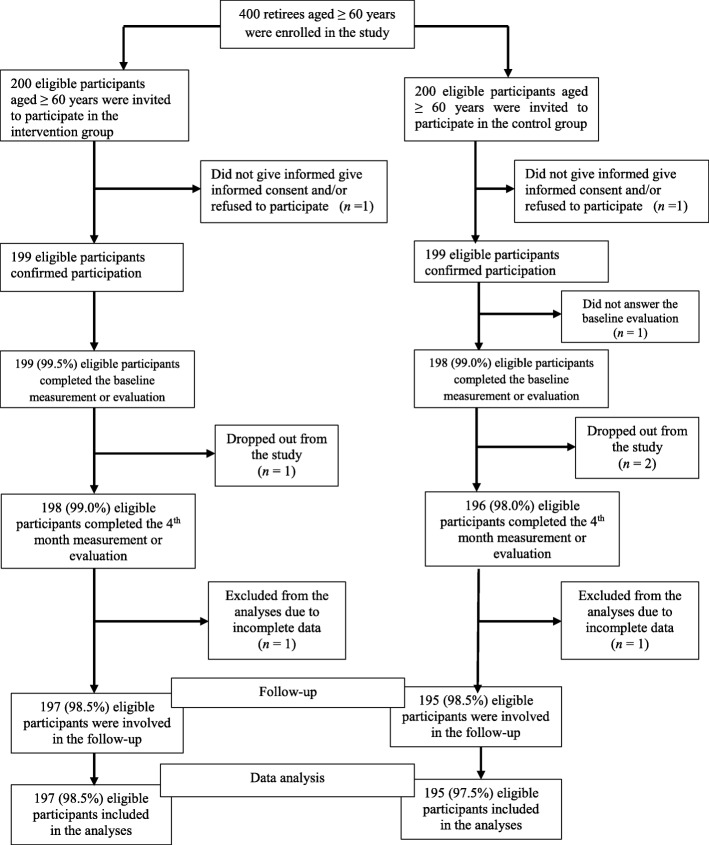
Flow diagram of participants and analysis. Depicts the flow diagram of participants and analysis. There were 200 participants each in the treatment and control groups (T-Group, *n* = 200; C-group, *n* = 200). Of these populations, 98.5% of participants in the intervention group and 97.5% of participants in the control group completed the study. The major reasons for refusing participation included lack of time/personal commitments, and illness. Incomplete data were also excluded from the analyses

We developed the health education intervention or programme to be culturally relevant. The health education programme on HT was developed based on the implementation research framework, the information-motivation-behavioral skills (IMB) model proposed by Fisher and Fisher [36]. The IMB model recognizes three fundamental contributing factors of the initiation and maintenance of health behaviors: accurate information that can be readily translated into health behavior performance; personal and social motivation to act on such information; and activity skills to carry out the health behavior positively and successfully. The IMB model explained the modifiable risk factors of HT such as behavior-related information and knowledge, beliefs and attitudes towards a particular health behavior and perceived social support, self-efficacy, and skills to negotiate preventive behaviors. To our knowledge, no published health education intervention on HT has used the IMB model to inform its design, content, content delivery, implementation and assessment of intervention effectiveness among older adults in south-east, Nigeria. We used a written script to standardize the contents of the intervention that included the meaning of hypertension, causes and risk factors for HT, signs and symptoms, measurement of BP and its interpretation, HT prevention, and self-care practices, and treatment and BP control. The themes of the 12-session intervention were designed based on the major topics. We conducted the sessions on the day of the week (Tuesdays for the intervention group and Thursdays for the control group) at a time the participants had considered the most convenient. The intervention lasted for 12 weeks (July to September, 2018), and each session lasted for about 60 min per week. We organized the sessions in groups of 50 retirees in both the intervention and control groups. The contents and educational materials used for the intervention were guided by the previous studies [37–42]. Health education experts and nurses delivered the contents of the educational programme to the treatment group retirees. The intervention materials included educational booklet on HT, videos, annotated charts and discussions covered all the topics. The videos were shown using a laptop and projector. The videos and annotated charts made it easier for those who were not strong in health literacy to understand the contents. This assisted the presenter to be consistent and provide detailed information. After each session, we gave a timeframe of 5 min for participants to ask questions. Correctness of the responses and answers to the questions raised by participants were validated by the health education experts and nurses. However, when questions asked by participants are outside the scope of the intervention materials, we counseled them to seek medical expertise. Also, the control group received health talk about HT and did not receive any intervention. Each session in the control group lasted for about 45 min. Only the health education experts delivered the health talk to the control group. We gave light refreshments (snacks and soft drinks) to the participants at the end of each session. We evaluated the HT knowledge, prevention and self-care practices of retirees in the intervention and control groups at the 4th and 5th months after the intervention (Fig. 1).

### Outcome variables

We used a four-part questionnaire based on the literature to collect data on participants’ demographic characteristics (age, gender, and level of education), and measure the HT knowledge [37–39], prevention, and self-care practices. We collected data in four steps. First, we conducted the face validity of the original 25-item version of the HT knowledge questionnaire/sub-scale of by five public health education experts with the aim of determining what to measure and what not to measure; second, a coefficient of those was completed; and third, a coefficient of ~ 0.5 was valid at 0.75, by the Items-Objective Congruence Index [43]. Secondly, the content validity of HT knowledge scale was evaluated through both quantitative and qualitative procedures. For the qualitative content validity assessment, 10 public health experts were interviewed and requested to evaluate the wording, readability, and clarity of the items, the accuracy of the dimensions and item distribution, and the correctness of the scoring protocol. The questionnaire was revised based on their observations. For the quantitative content validity assessment, 10 health education experts were invited to determine whether each item in the questionnaire was “necessary”, “suitable but not necessary”, or “not necessary at all”. The experts’ remarks were employed to compute the Content Validity Ratio (CVR). Based on the recommendation by Lawshe [44], items with a CVR of less than 0.62 were removed [45]. Subsequently, we required the experts to assess the relativity, simplicity, and clarity of the items by using a five-point Likert-type scale of “Not clear (1 point)”, “Fairly clear (2 points)”, “Clear (3 points)”, “Very clear (4 points)”, and “Absolutely clear (5 points)”. We calculated the Content Validity Index (CVI) for each item by dividing the number of experts who had scored that item 4 or 5 by the total number of the experts [46]. We considered the items with a CVI of greater than 0.78 as having good content validity [47]. After these procedures, five items were excluded from the questionnaire.

Furthermore, a pilot study was conducted to assess the construct validity of the 20-item HT knowledge questionnaire. We administered copies of the 20-item questionnaire to 100 retirees who were not included in the main study. The principal component analysis (PCA) was used to examine the construct validity of the 20-item HT knowledge questionnaire. Before performing the PCA, we assessed the suitability of the data for factor analysis. Inspection of the correlation matrix showed the presence of many coefficients of .30 and above. The Kaiser-Meyer Olkin value was .65 [48], and Bartlett’s Test of Sphericity reached statistical significance (*p* < .0001), supporting the factorability of the correlation matrix [49]. The PCA showed the presence of 6 components with eigenvalues exceeding 1.00, explaining 62.2% of the variance. An inspection of the scree plot showed a clear break after the second component. Using the Cattell’s scree test [50], it was decided to retain a two-factor solution component for further investigation. The two-factor component solution explained a total 33.5% of the variance, with component 1 contributing 23.7% and component 2 contributing 9.8%. The Oblimin rotation was performed to aid the interpretation of the results. The rotated solution showed the presence of simple structure [51], with both components showing some strong loadings and all variables loading substantially on only one component. There was a weak positive correlation between the two factors (*r* = .05). The items loading 0.40 and above were retained. Using the pattern matrix, a total of 15 items were retained [52] (Additional file 2: Table S1 and S2). The 15-item HT knowledge questionnaire had a good reliability (Kuder-Richardson-20 coefficient was 0.72) (Additional file 3). We validated the intervention using two pension zones (i.e. pilot study). The zones were not included in the main study. Five health education experts with Ph.D. and M.Ed. Degrees, and three nurses with a B.Sc. degree and adequate work experience (the nurses work in tertiary health institutions in Enugu) facilitated the baseline, post-intervention and follow-up data collection process. The baseline, post-intervention and follow-up data were collected under the supervisions of the principal investigators.

### Covariates

We collected the data on covariates which included the height and weight measurements for the body mass index (BMI) and blood pressure (BP) levels in the successive steps. We obtained written informed consent from the participants who fulfilled the inclusion criteria and were willing to participate in the study. The height and weight of all subjects were measured with an electronic digital scale at morning times between 10 a.m. to 11:30 a.m. under light clothes without shoes. The BP levels were measured from the right and left arms of the subjects in a sitting position by the nurses at the study site (the study site was agreed upon by the retirees). The BP was measured twice with 10 min interval. The systolic BP (SBP) and diastolic BP (DBP) were recorded at the first and fifth Korotkoff phases respectively using calibrated mercury sphygmomanometers. We used the average of the four BP measurements for analysis. Using the Joint National Committee Report-7 (JNC 7) classification of blood pressure for adults [53], we classified the subjects’ BP levels as follows: normal: SBP < 120 mmHg and DBP < 80 mmHg; PreHT: SBP 120–139 mmHg and/or DBP 80–89 mmHg; hypertension: SBP ≥ 140 mmHg and/or DBP ≥ 90 mmHg. The subjects who have BP measurement of SBP < 140 mmHg (≥ 60 age, SBP < 150 mmHg) and/or DBP < 90 mmHg at the time of study were classified as subjects with BP under control [40]. We considered the participants receiving antihypertensive treatment to have hypertension. The BMI was calculated by the formula (BMI = weight in kg/(height in m^2^) defined according to the WHO criteria [41]. Thus, we classified the participants as underweight (BMI = < 18.5 kg/m^2^), normal weight (BMI = 18.5–24.9 kg/m^2^), overweight (BMI = 25.0–29.9 kg/m^2^), obese class I (BMI = 30.0–34.9 kg/m^2^), obese class II (BMI = 35.0–39.9 kg/m^2^) or obese class III (≥ BMI 40 kg/m^2^). However, for ease of analysis, we categorized all the participants with BMI ≥ 30 as obese.

### Measures

#### Demographic variables

Demographic variables were assessed at the baseline. These included age (60–69 years, 70–79 years and ≥ 80 years), gender, height, weight, systolic and diastolic BP, level of education (primary, secondary, tertiary education or its equivalent).

##### Hypertension knowledge

The knowledge of HT was measured using a 15-item scale with a response format ‘True,’ ‘False,’ and ‘I don’t know.’ The 15 questions were as follows: one question about the meaning of HT; four questions about causes and risk factors for HT, one question about signs and symptoms; two questions about BP measurement and its interpretation, four questions about HT complications and three questions about HT treatment and BP control [37–39]. The knowledge index ranged from 0 to 15, with higher scores indicating greater HT knowledge post-intervention. We considered the mean HT knowledge scores at both the pre-intervention and post-intervention phases.

##### HT prevention and self-care practices

The HT prevention and self-care practices sub-scale consists of 15 questions that focused on six domains such as regular physical activities (PA), adequate and quality sleep, abstinence/reduction in use of addictive substances, healthy diet, medication adherence and home BP monitoring (HBPM).

##### Physical activity

We asked three questions about the participants’ PA using the Short International Physical Activity Questionnaire Form “last seven days recall” (IPAQ-SF) [54]. The IPAQ-SF asks respondents to indicate frequency and duration of walking, moderate intensity, vigorous-intensity and sitting activity performed for at least 10 min duration per session in the last seven consecutive day period [54, 55]. The PA categories may be examined independently to get the specific activity patterns or multiplied by their estimated value in Metabolic Equivalent of Tasks (METs) and summed to get an overall estimate of PA in a week [54–56]. This IPAQ-SF has been validated in Nigerian study [57]. We asked the participants about their PA levels in the past 7 days during the pre- and post-intervention phases. For instance, during the last 7 days, on how many days did you do vigorous physical activities like heavy lifting, digging, aerobics, or fast bicycling? During the last 7 days, on how many days did you do moderate physical activities like carrying light loads, bicycling at a regular pace, or doubles tennis? [54]. We excluded the item that elicits information on the total time spent sitting on a weekday. Participants were asked to indicate how much time (none, minutes and hours) they spent in doing such activities in the past 7 days. The PA measures used in this study included: minutes reported in vigorous, moderate, and walking activities and per week (Min week-1) and MET-minutes per week. We obtained the time spent in each activity category by multiplying the number of days per week with the minutes spent doing the activity per day and calculated the total weekly physical activity (MET-Min week-1) by multiplying the number of minutes spent in each activity category with the specific MET score for each activity. We made sure that the daily time spent on each of vigorous, moderate and walking activities ranged between 10 and 180 min for all participants. We used the MET intensity values to score IPAQ questions in this study. These included vigorous (8 METs), moderate (4 METs) and walking (3.3 METs) [54]. These categories followed the standard scoring criteria http://www.ipaq.ki.se. In this study, we assessed the PA using four scoring criteria as follows: none (0 points), walking METs of 3.3 (1 point), moderate activity with METs of 4 (2 points) and vigorous activity with 8 METs (3 points). The PA total score for the three items ranges from 0 to 9 points.

##### Quality and patterns of sleep

We assessed the quality and patterns of sleep using three items from the Pittsburgh Sleep Quality Index (PSQI) [58]. The PSQI is a self-reported questionnaire that measures sleep quality and patterns during the previous month and evaluates seven components of sleep: subjective quality, latency, duration, usual efficiency, sleep disturbances, medication use, and daytime dysfunction. A score > 5 is suggestive of a sleep disorder [58]. In this study, we used items #6 to #8 of the PSQI. For instance, the questions included: During the past month, how often have you taken medicine to help you sleep (prescribed or “over the counter”)? During the past month, how often have you had trouble staying awake while driving, eating meals, or engaging in social activity and during the past month, how much of a problem has it been for you to keep up enough enthusiasm to get things done? The items were assigned a Likert-type response format. Items #6 and #7 were assigned ‘Not during the past one month (0 point)’, ‘less than once a week (1 point)’, ‘once or twice a week (2 points)’, and ‘three or more times a week (3 points)’. However, item 8 was assigned ‘No problem at all’, ‘only a slight very problem’, ‘somewhat of a problem’, and ‘a very big problem’ [58]. The total score for the three items ranges from 0 to 9 points. However, we used an aggregate score of 3. A higher score on this scale is indicative of a sleep disorder, a lower score implies reduced sleep disorder, and a score of 0 suggests the absence of a sleep disorder.

##### Substance use abstinence/reduction

Substance use abstinence/reduction was assessed using three items. The first items included: have you ever smoked a cigarette before? This item was assigned a response option of ‘*Yes*’, ‘*No’, or ‘never smoked*’. The ‘*Yes*’ response was coded as 1 while the ‘*No’ and ‘never smoked’* responses were coded 0. Thus, a ‘Yes’ response to the question attracted 1 point while a ‘No’ or ‘never *smoked*’ response attracted 0 point. Alcohol use was measured using two items. For instance, ‘*how often do you have a drink containing alcohol in a day*, and *how many alcoholic drinks do you consume on average per week?’* The participants were asked to self-report how much alcohol they consumed on each drinking episode in the last 7 days. Since there is no Government recommended safe drinking guide in Nigeria, in that there are no alcohol policies [59], we asked the participants to describe their drinking in terms of the frequency of alcoholic drinks of various brands (beer, local gin, brandy, whiskey and other local alcoholic beverages) they had in the past 7 days. The first question was assigned a 3-point Likert-type scale of ‘never (0 point)’, ‘2-3 times a week (1 point)’, ‘3-4 times a week (2 points)’ and ‘≥ 6 times a week (3 points)’. The second question was also assigned a Likert-type response format of ‘none (0 point)’, ‘1-2 drinks (1 point)’, 3–5 drinks (2 points)’, and ‘≥ 6 drinks per week (3 points)’. Thus, the total score for this sub-scale ranges from 0 to 7 points. At the baseline, higher scores indicate harmful alcohol use. A score of 5–7 implies harmful alcohol use, a score of 1–4 implies moderate alcohol use and a score of *0* implies no substance use. Consequently, after the intervention, a score of 0 indicates no substance use or abstinence and a lower score indicates substance use reduction.

##### Healthy diet

We used one item that measures healthy diet from the dietary screening questionnaire (SDQ) for older adults developed by Bailey et al. [60]. The SDQ is a 24-h recalls for temporal distribution of dietary intake with 2 Dietary patterns. Dietary pattern 1 is consistent with a “prudent” dietary pattern, whereas dietary pattern 2 could be considered “Western” or low nutrient dense. Dietary pattern 1 was characterized by fruits, vegetables, lean white meat, dairy, and whole grain products (mainly cereals). Dietary pattern 2 contained foods that would be considered lower in nutrient density, such as sweets and candy, processed meats, and salty snacks [60]. However, we formulated the question based on the Dietary pattern 1. For instance, on the average, in that past 7 days, how often do you consume the following food groups (fresh fruits, vegetables, chicken, fresh fish, dairy, and whole grain products such as rice, corn, sorghum, millet, beans, whole-wheat bread, etc.)? The item was assigned a Likert-type format of ‘never (0 point)’, ‘1-2 times a week (1 point)’, ‘3-5 times a week (2 points)’, and ‘more than 6 times a week (3 points)’. The total score for this item ranges from 0 to 3 points. Higher scores indicate healthier diet.

##### Medication adherence

We used the 4-item Morisky Medication Adherence Scale (MMAS-4) or Morisky Scale [61–63] to assess medication adherence as a self-care practice in the two groups. The World Health Organization [64] defines adherence as the extent to which a person’s behaviour – taking medication, following a diet and/or executing lifestyle changes, corresponds with agreed recommendations from a health care provider. The MMAS-4 is the quickest to administer and score and is able to ascertain barriers to adherence due to its length [61]. For instance, *do you ever forget to take your anti-hypertensive/blood pressure medicine or other medicines prescribed by the doctor*? The structured question format with “yes” bias indicates non-adherence. The MMAS-4 has been validated in the broadest range of diseases and in patients with low literacy [62]. The MMAS consists of four items with a scoring protocol of “Yes” (0) and “No” (1). Total MMAS-4 scores range from 0 to 4 and have been categorized into three levels of adherence: high adherence (score = 0), medium adherence (score of 1 to 2), and low adherence (score 3 to 4) [58].

##### Home blood pressure monitoring (HBPM)

One of the most commonly recommended self-care practices for HT is the home blood pressure monitoring (HBPM). Evidence suggests that HBPM is “easy to perform, reliable, reproducible”, [65, 66], cost-effective [64] and reduces office visits, and the number of medications [66–68]. We assessed home blood pressure monitoring (HBPM) in this study using one item according to the National Institute for Clinical Excellence (NICE) guidelines [69]: *using the following guidelines, how often do you monitor or take measurement of BP at home?* The NICE guidelines for HBPM stipulate that when the HBPM is used to confirm a diagnosis of HT, it is essential to ensure that: 1) for each BP recording, two consecutive measurements are taken, at least 1 min apart with the person seated; 2) BP is recorded twice daily, ideally in the morning and evening; and 3) BP recording continues for at least 4 days, ideally for 7 days. The NICE guidelines for HBPM were outlined to guide the participants’ responses to the item. The guidelines were assigned a response format of “Never (0 point)”, rarely (1 point)’, ‘occasionally (2 points)’, and ‘always (3 points)’. Thus, the score index ranges from 0 to 9 points. However, we used an aggregate score of 3 points. Higher scores imply effective HBPM. Furthermore, the Cronbach’s alpha coefficient for the HT prevention and self-care practices scale was 0.84 (Additional file 4).

### Data processing and statistical analysis

We used the exploratory factor analysis (EFA) via the principal component analysis (PCA) and Oblimin rotation to validate the factor structure of the questionnaire as earlier explained. The EFA was conducted using the original 40-item questionnaire to determine whether the measures of construct were consistent with the authors’ comprehension of the nature of that construct. Ten items were excluded during data analysis due to low correlations. Thus, 30 items of the questionnaire were used for the study and included in the analysis. The comparisons of the demographic characteristics between the intervention/treatment and control groups at baseline were examined using independent samples *t*-test for continuous variables and Chi-square test for categorical variables. To examine the intervention effects on HT knowledge, prevention and self-care practices, one-way repeated measures analysis of variance (ANOVA) with time (baseline, 4th month and follow-up) as within-subjects factor and group (intervention/control) as between-subjects factor were performed. We conducted preliminary analyses to ensure that there was no infraction of the assumption of normality, linearity, homogeneity of variance and reliable measurement of the covariates. Missing measurements as a result of a participant’s withdrawal from the study were excluded from data analyses. Statistical significance was set at *p* < 0.05. The analyses were performed using the IBM SPSS 20.0 (IBM Corp., Armonk, NY, USA).

## Results

### Characteristics of the participants

We enrolled 400 participants in the study. The mean age of the participants was 65.9 (± 8.9) years, mean SBP and DBP were 136.5 (± 13.3) and 87.9 (± 9.1) respectively. The mean BMI was 23.9 (± 5.1) kg/m^2^. In total, majority of the participants was in age group 60–69 years (68.4), 50.3 and 49.7% of the sample were male and female respectively, and 55.9% had a tertiary education. The result further showed that more than half of the participants were hypertensive (55.6%) and majority had normal BMI (61.5%). The Chi-square tests showed that there were significant baseline differences between the groups based on gender and BMI categories. The independent samples t-tests showed that no significant differences were found in the mean SBP, DBP and BMI between the intervention group and control group (Table 1).

**Table 1:** Demographic characteristics of the sample at baseline

Characteristics	Total sample *(N =* 392)	Intervention group *(n* = 197)	Control group *(n* = 195)	*χ*^2^/*t*	*P*-value
Age, mean (SD)	65.9 (8.9)	65.6 (8.4)	66.2 (9.3)		
Age, *n* (%)				1.88	0.39
60–69 years	268 (68.4)	141 (71.6)	127 (65.1)		
70–79 years	95 (24.2)	43 (21.8)	52 (26.7)		
≥ 80 years	29 (7.4)	13 (6.6)	16 (8.2)		
Gender, *n* (%)				13.23	.000*
Male	197 (50.3)	81 (41.1)	116 (59.5)		
Female	195 (49.7)	116 (58.9)	79 (40.5)		
Educational level, *n* (%)				0.18	0.91
Primary education	56 (14.3)	29 (14.7)	27 (13.8)		
Secondary education	117 (29.8)	60 (30.5)	57 (29.2)		
Tertiary education	219 (55.9)	108 (54.8)	111 (50.7)		
Current BP					
SBP, mean (SD)	136.5 (13.3)	137.2 (12.9)	135.9 (13.5)	−0.88	0.38
DBP, mean (SD)	87.9 (9.1)	88.2 (9.9)	87.7 (8.1)	−0.45	0.65
BP Categories *n* (%)				3.61	0.17
Normal	41 (10.5)	15 (7.6)	26 (13.3)		
Pre-HT	133 (33.9)	67 (34.0)	66 (33.8)		
HT	218 (55.6)	115 (58.4)	103 (52.8)		
BMI, mean (SD)	23.9 (5.1)	23.8 (5.4)	23.9 (4.8)	0.04	0.97
BMI Categories, *n* (%)				8.64	0.04**
Underweight	38 (9.7)	27 (13.7)	11 (5.6)		
Normal	241 (61.5)	117 (59.4)	124 (63.6)		
Overweight	66 (16.8)	28 (14.2)	38 (19.5)		
Obese	47 (12.0)	25 (12.7)	22 (11.3)		

*HT* Hypertension, *BMI* Body Mass Index, *BP* blood pressure, *SBP* Systolic blood pressure, *DBP* Diastolic blood pressure. *P < .001; **P < 0.05

### Before and after the intervention differences in HT knowledge

Results of the paired comparison indicated that the mean HT knowledge of all the participants increased between the baseline (pre-test) and after the intervention (post-test). After the health education intervention, the mean HT knowledge score significantly increased in the T-group between baseline and 1 month (4th month) compared to those in the C-group (*p* < 0.0001) (Table 2).

**Table 2:** HT knowledge in the groups at baseline and after intervention

	Intervention group	Control group
	(*n* = 197)	(*n* = 195)
Time	Mean ± SD	Mean ± SD
Baseline	9.64 ± 3.35	9.69 ± 3.52
After intervention	12.63 ± 2.13	9.71 ± 3.47
Paired samples *T*-test results	*P* = 0.000*	*P* = 0.57

*P < 0.0001

### Before and after the intervention differences in HT prevention and self-care practices

Regarding the HT prevention and self-care practices (PSC), after the intervention, significant differences existed between the two groups. The paired samples analyses showed that PA increased significantly in the T-group, whereas no increase was found the in C-group between baseline and after intervention (*P* = 0.007). Additionally, sleep pattern and quality (*P* = 0.003), substance use abstinence (*P* = 0.000), healthy diet (*P* = 0.000), and medication adherence (*P* = 0.000) improved significantly in the T-group between baseline and 1 month after intervention, whereas the control remained similar, decreased or slightly increased between baseline and 1 month after intervention (Table 3). However, no significant difference was found for HBPM between the two groups 1 month after the intervention ((*P* = 0.21) (Table 3).

**Table 3:** Dimensions of HT prevention and self-care practices in treatment and control groups at baseline and after the intervention

Time	Dimensions of HT prevention & self-care practices	Intervention group Mean ± SD	Control group Mean ± SD	*Test results *P*-value
Baseline				
	Physical activity	1.92 ± 0.97	1.90 ± 1.01	0.76
	Sleep pattern & quality	1.87 ± 1.10	1.95 ± 0.93	0.10
	Substance use abstinence	2.29 ± 0.92	2.15 ± 0.83	0.51
	Healthy diet	0.76 ± 0.43	0.72 ± 0.45	0.08
	Medication adherence	2.27 ± 1.18	2.26 ± 1.66	0.07
	HBPM	1.42 ± 0.83	1.57 ± 0.74	0.49
After intervention				
	Physical activity	2.15 ± 0.81	1.90 ± 1.02	0.007**
	Sleep pattern & quality	1.57 ± 1.04	1.95 ± 0.95	0.003**
	Substance use abstinence	1.68 ± 1.02	2.10 ± 0.94	0.000*
	Healthy diet	1.27 ± 0.65	0.74 ± 0.44	0.000*
	Medication adherence	1.86 ± 1.06	2.44 ± 0.83	0.000*
	HBPM	1.51 ± 0.69	1.57 ± 0.65	0.21

*Paired samples t-test. HBPM, Home blood pressure monitoring

*P < 0.0001; **P < 0.001

### Intervention effects on HT knowledge, prevention and self-care practices

The one-way repeated measures ANOVA analyses showed statistically significant effects (between-groups analysis) for HT knowledge (*P* = 0.000, ηp2 = 0.36), physical activity (*P* = 0.000, ηp2 = 0.06), sleep pattern and quality (*P* = 0.000; ηp2 = 0.08), substance use abstinence (*P* = 0.000; ηp2 = 0.21), healthy diet (*P* = 0.000; ηp2 = 0.30), medication adherence (*P* = 0.000; ηp2 = 0.28), and HBPM (*P* = 0.000; ηp2 = 0.10) with small to large effect sizes [70] (Table 4) (Additional files 5, 6, 7, 8, 9 and 10). The effect sizes ranged from 0.06 to 0.36, showing that the intervention effects are both limited and substantial practical significance. The paired samples analyses indicated that HT knowledge significantly increased between baseline and 2-month follow-up in T-group compared to the control group (*P* = 0.000). A similar result was obtained for PA between baseline and 2-month follow-up (*P* = 0.000). Additionally, PSC such as physical activity (*P* = 0.000), sleep pattern and quality (*P* = 0.000), substance use abstinence (*P* = 0.000), healthy diet (*P* = 0.000), medication adherence (*P* = 0.000), and HBPM (*P* = 0.000) significantly improved between baseline and 2-month follow-up in the T-group than C-group (Table 4). The one-way repeated measures ANOVA analyses showed significant time-by-group interaction effects (within-groups) for HT knowledge (*P* = 0.000, ηp2 = 0.34), physical activity (*P* = 0.000, ηp2 = 0.04), sleep pattern and quality (*P* = 0.000; ηp2 = 0.06), substance use abstinence (*P* = 0.000; ηp2 = 0.16), healthy diet (*P* = 0.000; ηp2 = 0.28), medication adherence (*P* = 0.000; ηp2 = 0.26), and HBPM (*P* = 0.000; ηp2 = 0.04) with small to large effect sizes [70] (Table 5) (Additional files 5, 6, 7, 8, 9 and 10).

**Table 4:** Intervention effects on HT knowledge, prevention and self-care practices

Measures	Baseline Mean ± SD	After intervention (4th month) Mean ± SD	Follow-up (5th month) Mean (SD)	*F*	ηp2	Paired analyses
						Baseline & follow-up *P*-value
HT knowledge						
Intervention group	9.64 ± 3.35	12.63 ± 2.13	13.13 ± 1.39	108.12*	0.36	0.000*
Control group	9.69 ± 3.52	9.71 ± 3.47	9.76 ± 3.42			0.16
HT PSC						
Physical activity						
Intervention group	1.92 ± 0.97	2.15 ± 0.81	2.06 ± 0.88	12.71*	0.06	0.000*
Control group	1.90 ± 1.01	1.90 ± 1.02	1.91 ± 0.99			0.59
Sleep pattern and quality						
Intervention group	1.87 ± 1.10	1.57 ± 1.04	1.41 ± 0.99	17.75*	0.08	0.000*
Control group	1.95 ± 0.93	1.95 ± 0.95	1.92 ± 0.93			0.05
Substance use abstinence						
Intervention group	2.29 ± 0.92	1.68 ± 1.02	1.20 ± 0.98	52.67*	0.21	0.000*
Control group	2.15 ± 0.83	2.10 ± 0.94	2.06 ± 0.95			0.28
Healthy diet						
Intervention group	0.76 ± 0.43	1.27 ± 0.65	1.63 ± 0.82	81.98*	0.30	
Control group	0.72 ± 0.45	0.74 ± 0.44	0.74 ± 0.45			0.10
Medication adherence						
Intervention group	2.27 ± 1.18	1.86 ± 1.06	0.91 ± 0.94	75.19*	0.28	0.000*
Control group	2.26 ± 1.66	2.44 ± 0.83	2.27 ± 1.14			0.08
HBPM						
Intervention group	1.42 ± 0.83	1.51 ± 0.69	1.78 ± 0.77	20.88*	0.10	0.000*
Control group	1.57 ± 0.74	1.57 ± 0.65				0.15

*HT* hypertension, *PSC* Prevention and self-care, *HBPM* home blood pressure monitoring

*F* Wilks’ lambda F value, *ηp2* partial eta squared

*P < 0.0001

**Table 5:** Intervention effects (time by group interaction) on HT knowledge, prevention and self-care practices

Measures	Baseline Mean ± SD	After intervention (4th month) Mean ± SD	Follow-up (5th month) Mean (SD)	*F* Time by group	ηp2
HT knowledge					
Intervention group	9.64 ± 3.35	12.63 ± 2.13	13.13 ± 1.39	98.54*	0.34
Control group	9.69 ± 3.52	9.71 ± 3.47	9.76 ± 3.42		
HT PSC					
Physical activity					
Intervention group	1.92 ± 0.97	2.15 ± 0.81	2.06 ± 0.88	8.09*	0.04
Control group	1.90 ± 1.01	1.90 ± 1.02	1.91 ± 0.99		
Sleep pattern and quality					
Intervention group	1.87 ± 1.10	1.57 ± 1.04	1.41 ± 0.99	12.76*	0.06
Control group	1.95 ± 0.93	1.95 ± 0.95	1.92 ± 0.93		
Substance use abstinence					
Intervention group	2.29 ± 0.92	1.68 ± 1.02	1.20 ± 0.98	37.94*	0.16
Control group	2.15 ± 0.83	2.10 ± 0.94	2.06 ± 0.95		
Healthy diet					
Intervention group	0.76 ± 0.43	1.27 ± 0.65	1.63 ± 0.82	74.66*	0.28
Control group	0.72 ± 0.45	0.74 ± 0.44	0.74 ± 0.45		
Medication adherence					
Intervention group	2.27 ± 1.18	1.86 ± 1.06	0.91 ± 0.94	69.16*	0.26
Control group	2.26 ± 1.66	2.44 ± 0.83	2.27 ± 1.14		
Home blood pressure monitoring					
Intervention group	1.42 ± 0.83	1.51 ± 0.69	1.78 ± 0.77	8.24*	0.04
Control group	1.57 ± 0.74	1.57 ± 0.65			

*HT* hypertension, *PSC* Prevention and self-care, *F* Wilks’ lambda F value, *ηp2* partial eta squared

*P < 0.0001

## Discussion

The results of our study showed that the educational intervention had a positive effect on increasing retirees’ HT knowledge, and any other individual outcome. Our findings have contributed to limited data on the effectiveness of health education interventions in sub-Saharan Africa. In the T-group, the mean HT knowledge significantly increased between baseline and 1 month (4th month) compared to the C-group. Adequate knowledge of HT and its prevention has been identified as a precondition for lifestyle modifications, medication adherence and effective control of BP among hypertensive patients and older adults [22, 23]. This finding is consistent with a previous study that reported higher post-test scores on hypertension knowledge, attitude and practices (KAP) than the pre-test scores [71]. Several studies have indicated the prevalence of poor knowledge about HT among older adults [19, 20, 24]. Therefore, valid and culturally contextualized health education intervention on HT is vital for increasing knowledge among the hypertensive and non-hypertensive older adults. Implementation of health education interventions that target knowledge improvement among older adults in diverse regions in Nigeria may reduce the prevalence of HT and its associated disability, mortality and morbidities in this sub-population. However, evidence suggests knowledge does not always lead to the desired behavioural change [72].

According to the study findings, HT prevention and self-care practices such as PA, sleep pattern and quality, substance use abstinence, healthy diet, and medication adherence increased or improved significantly in the T-group compared with the control group between baseline and 1 month after intervention. The present study shows that a culturally sensitive and well-organized model-based educational intervention has the potential to improve prevention and self-care practices of older adults. Thus, the study validates the beneficial effects of health education interventions on lifestyle modification among older adults. The findings are compatible with previous studies that reported improvement in self-care practices of HT in older patients [71, 73]. However, no significant difference was found for HBPM between the two groups at 1 month after the intervention. Some plausible reasons may explain this finding. Firstly, unavailability of a BP monitoring device due to its cost and lack of the required skills for effective use may be implicated. Evidence suggests that the use of auscultatory devices (mercury, aneroid or other) is not recommended for HBPM because their use requires the presence of a trained observer while BP monitors that use the oscillometric method are recommended [65, 68]. However, the cost of BP monitoring devices in Nigeria seems too exorbitant for the poor. For instance, the M2 basic automatic blood pressure and the OMRON M7 intelli IT are sold for about #14,000 ($38.4, i.e. #365/USD) and #35,000 ($95.9) respectively in the pharmaceutical stores or retails. Due to economic downturn, many retirees in Nigeria do not receive their monthly stipends regularly. Therefore, some retirees may not have the money to buy the BP monitors. Hence, it becomes imperative for policy makers, and government to fully subsidize health care products such as BP monitors for older adults. In addition, older adults including retirees should be given free BP monitors at health facilities. A lack of the necessary skills is another potential factor that may explain this finding. For instance, evidence suggests that effective HBPM requires the hearing acuity of the operator, the sensitivity of the stethoscope, positioning of the cuff bladder center is directly above the brachial artery, and validation of the automatic electronic BP monitors before use [74, 75]. Although, we provided demonstrations on self-BP measurements during the intervention, it is possible that some retirees did not acquire enough skills in the short-term. The one-month post intervention period may probably be too short for participants to observe full uptake of HBMP. Thus, convincing effects of the intervention HBPM may not be expected in the short-term period. This finding contradicts a previous study [73]. Further research is needed to investigate whether retirees who received long-term educational intervention on HT have improved HBPM outcomes.

Furthermore, considering the underlying constructs of IMB model [36, 73], the intervention focused on increasing HT knowledge and improving prevention and self-care practices of HT. The conceptualization of the IMB model holds that information, motivation, and behavioral skills are the fundamental determinants of a preventive behavior [36, 73]. Although, the IMB was developed to predict HIV preventive behavior and necessary components of HIV prevention intervention, it has been applied to other areas [76, 77]. The post-intervention and follow-up (i.e., 5th month) effects of the intervention were significant. The follow-up intervention was meant to reinforce the key outcomes of the study in the T-group. After evaluation of the primary outcomes at 1-month post-intervention, we observed the need for a follow-up. After the follow-up, the participants’ HT knowledge increased considerably and there were significant changes in the HT prevention and self-care practices. This reflected in the significant between- and within-group effects observed in the T-group compared with the C-group. This implies that the intervention effects are of considerable practical significance for public health. This finding is compatible with previous studies [71, 73]. This finding suggests the need for the implementation of more cost-effective and culturally sensitive educational interventions among older adults in Nigeria. Educational interventions use behavioural change techniques (e.g., provision of personal feedback, goal-setting, and self-monitoring of behaviour etc.) that facilitate maintenance or sustenance of health behaviours [27]. Furthermore, compared to controlled clinical trials, educational interventions may be more accessible to hard-to-reach populations (e.g. people in isolated communities or creeks in the Niger Delta region) in Nigeria.

### Strength and limitations

The strength of this study was the use of a quasi-experimental design to determine the effectiveness of a health education intervention delivered in a community setting. The quasi-experimental (non-randomized) studies are increasingly adopted to evaluate population health interventions by health experts [78, 79]. Furthermore, the findings of such studies are probably useful to policymakers when the intervention is conducted under real-life situations without manipulation for research [80]. The findings of the study are also consistent with previous research [71–73]. Also, the intervention was theory based and integrated cogent constructs of IMB model. To our knowledge, this is the first study assessing the effects of an IMB model-based health education intervention on HT knowledge and other individual outcomes among older adults in south-east, Nigeria.

However, the study has several limitations. One major limitation is the potential effects of external factors or interventions unconnected to the current intervention. For instance, life events such as onset of illness, disability, and involvement in other intervention in a health facility might have influenced participants’ responses either positively or negatively. Another limitation of the study is the relatively small sample size. Although, the *priori* power analysis showed that our sample was adequate for the study; however, a relatively small sample size makes our study more exposed to the potentially confounding effects of life events. For instance, it is likely that there was an improvement in a retiree’s HT knowledge, prevention and self-care practices after the intervention and follow-up, this improvement may be invalidated by adverse life events such as death of a family member. The use of larger samples and more rigorous study design such as randomized controlled trials (RCTs) in future studies would mitigate the effects of potentially confounding variables.

The study timeframe is another limitation. The length of time for substantial change in the HT prevention and self-care practice was relatively short. The follow-up period (i.e., 2 months after the intervention) might not be enough. Therefore, the long-term effects of the intervention could not be assessed. For instance, there was a marginal improvement in the participants’ PA 1 month after intervention, and a slight reduction in PA score 2 months after the intervention. Thus, longer follow-up (e.g. 6 months or 1 year) is necessary to assess maintenance of PSC practices in future studies.

In this study, there were significant baseline differences between the groups based on gender and BMI categories. Also, there are more women in the T-group compared to the C-group. The observed gender difference in the study might have influenced the study outcomes. Although, we tried to ensure gender balance in the allocation of participants to both the T-group and C-group, the dropouts were predominantly males. Evidence suggests [81] that significant gender differences exist in some aspects of therapy, coping behaviour, and help-seeking. Since women are known to be more concerned with their health than men, they could also be more responsive than men to a health education intervention designed to improve their HT knowledge, prevention and self-care practices. Thus, the success of future health interventions could improve greatly if gender of participants is considered and gender balance is ensured by researchers. Future studies may benefit from interventions designed with features to attract and retain male participants and reduce dropout rates. Furthermore, there were BMI differences in both groups at the baseline and the differences might have impacted the study results. Nevertheless, we believe that the random assignment of participants to both groups would have reduced the effect of participants’ BMI on the study outcomes. Future studies employing RCTs might investigate the effect of participants’ BMI on similar health interventions.

We used self-report measures in this study, which may introduce response bias. However, we believe that the use of well-validated scales would minimize the bias. Additionally, this study only included retirees in Enugu state, so the results may not be generalizable to other older adults who retired from the federal civil service or private firms, and those who are self-employed. This may have also limited its generalizability. Repetition of this study with other categories of older adults in diverse settings and communities in Nigeria would thus be needed. We also observed few dropouts in both groups. Dropout has been identified to limit the generalizability of findings [27]. It also suggests that some aspects of the intervention may be too arduous or time-consuming for the participants. Therefore, future research should tackle the exact reasons for dropout.

## Conclusion

Overall, our findings provide evidence for the efficacy of a community-based health education intervention in retirees and validated the importance of adopting IMB model in this group. Hence, a community-based health education intervention/programme targeted at older adults can increase HT knowledge, improve prevention and self-care practices at the population level. Also, further research is needed to develop preventive health interventions based on the Information-Motivation-Behavioral (IMB) Skills model to increase knowledge about HT, promote prevention and self-care behaviours among Nigerian retirees in diverse settings.

## Additional files

Additional file 1: Results of the statistical power analysis using G power software. (DOCX 75 kb)

Additional file 2: Results of exploratory factor analysis. (XLS 81 kb)

Additional file 3: Cronbach alpha reliability test for hypertension knowledge scale. (XLS 34 kb)

Additional file 4: Cronabch alpha reliability test for HT prevention & self-care practices scale. (XLS 34 kb)

Additional file 5: Results of one-way repeated measures ANOVA for HT knowledge T1-T3. (XLS 39 kb)

Additional file 6: Results of one-way repeated measures ANOVA for HBPM T1-T3. (XLS 39 kb)

Additional file 7: Results of one-way repeated measures ANOVA for healthy diet T1-T3. (XLS 39 kb)

Additional file 8: Results of one-way repeated measures ANOVA for medication adherence T1-T3. (XLS 39 kb)

Additional file 9: Results of one-way repeated measures ANOVA for PA T1-T3. (XLS 39 kb)

Additional file 10: Results of one-way repeated measures ANOVA for Sleep quality T1-T3. (XLS 39 kb)

Additional file 11: Results of one-way repeated measures ANOVA for substance use T1-T3. (XLS 39 kb)

Additional file 12: Results of paired samples T-tests. (DOCX 20 kb)

## Abbreviations

ANOVA: Analysis of Variance
BMI: Body Mass Index
CVD: Cardiovascular Disease
DBP: Diastolic Blood Pressure
HBPM: Home Blood Pressure Monitoring
HT: Hypertension
IMB: Information-Motivation-Behavioral Skills Model
IPAQ-SF: Short International Physical Activity Questionnaire Form
LGAs: Local Government Areas
METs: Metabolic Equivalent of Tasks
MMAS-4: 4-item Morisky Medication Adherence Scale
NUP: Nigeria Union of Pensioners
PA: Physical Activity
PCA: Principal Component Analysis
PSC: Prevention and Self-care
PSQI: Pittsburgh Sleep Quality Index
SBP: Systolic Blood Pressure
SDQ: Dietary Screening Questionnaire
