# Supplementary material for: Effects of a health education intervention on hypertension-related knowledge, prevention and self-care practices in Nigerian retirees: a quasi-experimental study
Source: Arch Public Health. 2019 May 23;77:23. doi: 10.1186/s13690-019-0349-x (PMC6532220; doi:10.1186/s13690-019-0349-x)
Supplement: Supplementary file 1 — Results of the statistical power analysis using G power software. (DOCX 75 kb) [file 13690_2019_349_MOESM1_ESM.docx]

**Additional file 1.** Results of statistical power analysis

**Fig 1**. Central and noncentral distributions

**Supplemental Table 1**. Protocol of power analyses

**t tests -** Means: Difference between two independent means (two groups)

**Analysis:** A priori: Compute required sample size

**Input:** Tail(s) = Two

Effect size d = 0.3333333

α err prob = 0.05

Power (1-β err prob) = 0.80

Allocation ratio N2/N1 = 1

**Output:** Noncentrality parameter δ = 2.8185888

Critical t = 1.9683522

Df = 284

Sample size group 1 = 143

Sample size group 2 = 143

Total sample size = 286

Actual power = 0.8020829

**Fig 2**. X-Y plot
