# Supplementary material for: Effects of a health education intervention on hypertension-related knowledge, prevention and self-care practices in Nigerian retirees: a quasi-experimental study
Source: Arch Public Health. 2019 May 23;77:23. doi: 10.1186/s13690-019-0349-x (PMC6532220; doi:10.1186/s13690-019-0349-x)
Supplement: Supplementary file 12 — Results of paired samples T-tests. (DOCX 20 kb) [file 13690_2019_349_MOESM12_ESM.docx]

**Additional file 12**

**CONSORT 2010 checklist of information to include when reporting a randomized trial**

| **Section/Topic** | **Item**  **No** | **Checklist item** | **Reported**  **on page No** |
| --- | --- | --- | --- |
| Title and abstract | 1a | Identification as a randomized trial in the title | Title Page |
|  | 1b | Structured summary of trial design, methods, results, and conclusions | Abstract |
| **Introduction** |  |  |  |
| Background and  objectives | 2a | Scientific background and explanation of rationale | Introduction  P3-4. |
|  | 2b | Specific objectives or hypotheses | Introduction.  P5-6. |
| **Methods** |  |  |  |
| Trial design | 3a | Description of trial design (such as parallel, factorial) including allocation ratio | Study design, setting and population  P6-9. |
|  | 3b | Important changes to methods after trial commencement (such as eligibility criteria), with reasons | *NA |
| Participants | 4a | Eligibility criteria for participants | Study design, setting and population. P7. |
|  | 4b | Settings and locations where the data were collected | P6. |
| Interventions | 5 | The interventions for each group with sufficient details to allow replication, including how and when they were actually administered | P8. |
| Outcomes | 6a | Completely defined pre-specified primary and secondary outcome measures, including how and when they were assessed | P9-15. |
|  | 6b | Any changes to trial outcomes after the trial commenced, with reasons | *NA |
| Sample size | 7a | How sample size was determined | P6-7 |
|  | 7b | When applicable, explanation of any interim analyses and stopping guidelines | *NA |
| Randomisation: |  |  |  |
| Sequence | 8a | Method used to generate the random allocation sequence | *NA |
| generation | 8b | Type of randomisation; details of any restriction (such as blocking and block size) | *NA |
| Allocation  concealment  mechanism |  | Mechanism used to implement the random allocation sequence (such as sequentially numbered containers), describing any steps taken to conceal the sequence until interventions were assigned | *NA |
| Implementation | 10 | Who generated the random allocation sequence, who enrolled participants, and who assigned participants to interventions |  |
| Blinding | 11a | If done, who was blinded after assignment to interventions (for example, participants, care providers, those assessing outcomes) and how | *NA |
|  | 11b | If relevant, description of the similarity of interventions | *NA |
| Statistical methods | 12a | Statistical methods used to compare groups for primary and secondary outcomes | P16-17 |
|  | 12b | Methods for additional analyses, such as subgroup analyses and adjusted analyses | P16-17 |
| **Results** |  |  |  |
| Participant flow (a  diagram is strongly  recommended) | 13a | For each group, the numbers of participants who were randomly assigned, received intended treatment, and were analysed for the primary outcome | See Figure 1 |
|  | 13b | For each group, losses and exclusions after randomization, together with reasons | See Figure 1 |
| **Recruitment** | 14a | Dates defining the periods of recruitment and follow-up | P7. |
|  | 14b | Why the trial ended or was stopped | See Figure 1 |

*Note*. *NA, Not applicable

**CONSORT 2010 checklist of information to include when reporting a randomized trial (continued)**

| **Section/Topic** | **Item**  **No** | **Checklist item** | **Reported**  **on page No** |
| --- | --- | --- | --- |
| Baseline data | 15 | A table showing baseline demographic and clinical characteristics for each group | Table 1  P18. |
| Numbers analysed | 16 | For each group, number of participants (denominator) included in each analysis and whether the analysis was by original assigned groups | Table 2 & Results  P18. |
| **Outcomes and**  **estimation** | 17a | For each primary and secondary outcome, results for each group, and the estimated effect size and its precision (such as 95% confidence interval) | Tables 3-5. & Results  P18-22. |
|  | 17b | For binary outcomes, presentation of both absolute and relative effect sizes is recommended | Tables 3-5 & Results  P18-22 |
| Ancillary analyses | 18 | Results of any other analyses performed, including subgroup analyses and adjusted analyses, distinguishing pre-specified from exploratory | Table S1.  Table S2. |
| Harms | 19 | All important harms or unintended effects in each group (for specific guidance see CONSORT for harms) | *NA |
| **Discussion** |  |  |  |
|  | 20 | Trial limitations, addressing sources of potential bias, imprecision, and, if relevant, multiplicity of analyses | Discussion.  P25-26. |
| Generalisability | 21 | Generalisability (external validity, applicability) of the trial findings | Discussion.  P22-24. |
| Interpretation | 22 | Interpretation consistent with results, balancing benefits and harms, and considering other relevant evidence | Discussion.  P22-24. |
| **Other information** |  |  |  |
| Registration | 23 | Registration number and name of trial registry | P27-28. |
| Protocol | 24 | Where the full trial protocol can be accessed, if available | *NA |
| Funding | 25 | Sources of funding and other support (such as supply of drugs), role of funders | *NA |

*Note*. *NA, Not applicable
